# Colored Surfaces Made of Synthetic Eumelanin

**DOI:** 10.3390/nano11092320

**Published:** 2021-09-07

**Authors:** Gema Marcelo, María del Mar López-González, Milena Vega, Carlos Pecharromán

**Affiliations:** 1 Departamento de Química Analítica, Química Física e Ingeniería Química, Universidad de Alcalá, 28805 Alcalá de Henares, Spain; 2 Instituto de Investigación Química “Andrés M. Del Rio” (IQAR), Universidad de Alcalá, 28805 Alcalá de Henares, Spain; 3 Instituto de Ciencia y Tecnología de Polímeros (ICTP, CSIC), C/Juan de la Cierva 3, 28006 Madrid, Spain; mar@ictp.csic.es; 4 Departamento de Ingeniería Química y Textil, Universidad de Salamanca, Pl/La Merced s/n, 37008 Salamanca, Spain; mvega@usal.es; 5 Instituto de Ciencia de los Materiales de Madrid (ICMM, CSIC), C/Sor Juana Inés de la Cruz 3, 28049 Madrid, Spain

**Keywords:** color, eumelanin, interference, refractive index, surface

## Abstract

The polymerization of 3,4-dihydroxy-L-phenylalanine leads to a carboxylic acid-rich synthetic melanin-like material (poly-L-DOPA). Synthetic melanin most resembles natural eumelanin in chemical structure. However, its deposition on surfaces leading to colored surfaces by interference is not as easy to accomplish as in the case of the preparation of colored surfaces by dopamine hydrochloride polymerization. This study deals with the preparation of new colored surfaces made from poly-L-DOPA displaying vivid colors by interference. These surfaces were obtained by depositing thin films of poly-L-DOPA on a reflective silicon nitride substrate. A high ionic strength in the polymerization medium was essential to accomplish the coating. The effect of ionic strength on the resulting surfaces was studied via reflectance, Atomic Force Microscopy (AFM) and Scanning Electron Microscopy (SEM). The refractive index was determined by ellipsometry, and was nearly constant to 1.8 when *λ* > 650 nm. In the visible spectral region, the imaginary part of the refractive index becomes relevant. The refractive index in the visible wavelength range (400–600 nm) was in the range 1.7–1.80.

## 1. Introduction

Many colors in nature are the result of the interaction of light with structured materials [1]. Interference, diffraction, or the selective reflection of light from these structures are the main underlying physical phenomena. Thin-film interference is perhaps the simplest source of structural color [2]. It is observed when an incident light wave is reflected by each boundary of a thin film, and the two reflected waves interfere constructively to form a new wave. In the case that absorption and interference combine, the color palette becomes more visually pleasant [3]. Such is the case of the striking colors that can be observed in some bird feathers due to the structuring of melanin pigments [4,5]. Melanins are natural pigments with interesting optical properties. They have a broadband monotonic absorption in the entire UV-visible range in addition to a large refractive index [6]. In this sense, by a clever use of these properties, new biomimetic photonic materials, with variable response at different wavelengths, may be synthetically manufactured [6].

To date, dopamine hydrochloride has a central role in the preparation of synthetic melanins. It spontaneously polymerizes in a basic aqueous medium to produce insoluble pigments with similar properties to its natural analogous form. Similarly, polydopamine is able to coat a wide variety of substrates [7,8]. Thereby, polydopamine colored surfaces have been demonstrated by using different substrates, and the colors were simply tuned by varying the polydopamine thickness [9,10,11]. In a recent work [12], a methodology to generate colored surfaces was reported by depositing a polydopamine ultra-thin film on a reflective silicon nitride substrate. In this work, the authors established that when a reflective substrate is used, only a small polydopamine deposit of 20 nm in thickness was needed to obtain uniform blue surfaces. As a result, uniform color surfaces concomitant in thickness were used to determine the refractive index of polydopamine by ellipsometry spectroscopy.

On the other hand, colored melanin surfaces obtained from the polymerization of 3,4-dihydroxy-L-phenylalanine (DOPA) and, consequently, the characterization of the poly-L-DOPA refractive index are seldom explored. DOPA is an important free amino acid that is the initial precursor in eumelanin biosynthesis [13]. However, outside living conditions, the oxidation of DOPA in aqueous solutions leads to a carboxylic acid-rich synthetic melanin-like material (poly-L-DOPA) that is highly soluble in water [14,15], unlike polydopamine that is a carboxylic-free acid pigment. Therefore, poly-L-DOPA is the most similar synthetic melanin to our eumelanin in chemical structure. The repulsion of the negatively charged carboxylic group seems to hinder the aggregation of the poly-L-DOPA oligomers, making the surface coating unsuccessful. For this reason, the use of poly-L-DOPA to design photonic materials has not been reported.

Importantly, it has been recently reported that poly-L-DOPA coating over different surfaces can be achieved when DOPA oxidative polymerization was conducted in an aqueous medium of high ionic strength [16]. With this result in mind, the objective of this work was to prepare surfaces made of poly-L-DOPA, displaying vivid colors by interference. These surfaces were obtained by depositing thin films of poly-L-DOPA on a reflective silicon nitride substrate. The procedure was conducted by immersing the silicon nitride wafer in a DOPA polymerization medium (pH = 8.5) that has a high ionic strength (provided by sodium chloride). The effect of salt concentration on the poly-L-DOPA deposition was studied. The quality of the deposit could easily be perceived by the homogeneous color that these deposits present. The thin films were characterized by scanning electron and atomic force microscopies to estimate the thickness and roughness. Finally, the characterization of surfaces by ellipsometry spectroscopy permitted the determination of the optical response and, more specifically, the refractive index of poly-L-DOPA. Additionally, the accurate determination of the eumelanin refractive index could be an advance in medicine. Anormal refractive index areas are investigated in skin examination for the diagnosis of lesions by using non-invasive optical imaging techniques, such as reflectance confocal microscopy. Precise determination of the refractive index can help to establish new models or methods that can assist the diagnosis in early stages [17].

## 2. Materials and Methods

The AFM images (5 µm × 5 µm) were obtained with a Veeco Multimode scanning probe microscope equipped with a Nanoscope IV (Veeco Instruments, Santa Barbara, CA, USA), a controller operating in tapping mode with a phosphorus doped silicon cantilever (model RTESP). Mean square roughness values were extracted from these images. The surface images were obtained by scanning electron microscopy (SEM) using a Hitachi SU-8000 instrument (Hitachi, Ltd., Tokyo, Japan). Raman spectra were obtained with a Micro-Raman Spectrometer (LabRAM HR Evolution; Horiba, Kyoto, Japan). Surfaces were excited at 785 nm. Optical characterization: both ellipsometric and reflectometric measurements were taken with a GES 5E (SOPRA Company, Courbevoie, France), provided with a variable angle goniometer, Xe-lamp, monochromator and photomultiplier. The reflectance diffuse spectrum was characterized in a Jasco 616 spectrophotometer (Jasco Co., Tokyo, Japan).

### 2.1. Silicon Wafer Characteristics

Crystalline silicon wafers with a thickness of 520 µm and coated with a 200 nm layer of silicon nitride (LPCVD) were purchased from Addison Engineering, Inc. The characteristics of the wafer were as follows: (i) front finish: mirror polish; (ii) back finish: etch; (iii) type: p/Boron; and (iv) orientation: (100).

### 2.2. Poly(L-DOPA) Coating Process

*Time effect:* The silicon wafer piece of ca 1 cm × 1 cm was immersed in 30 mL of a buffered solution of L-DOPA at pH 10 (Merck, >98%) (2 mg/mL). The buffer was trizma-HCl (Sigma-Aldrich, Darmstadt, Germany) (50 mM, pH 8.5) and contained NaCl at a concentration of 0.87 M. The polymerization was left to proceed at room temperature in an open flask with controlled magnetic agitation (200 rpm) at different times: 2, 3, 4, 5, 6, 7, 8 and 9 h. After each time, the wafer was washed with deionized water and air dried. The poly-L-DOPA aggregate dispersion was dialyzed (MWCO = 6000–8000) against water for 5 days in order to remove the buffer medium and remaining monomer molecules. After dialysis, the UV-vis absorption spectrum of the poly-L-DOPA dispersion in water was characterized. *NaCl concentration effect:* The same procedure as described above was used. However, coating was studied at 9 h of polymerization, while the NaCl concentration in the buffer was varied. The following NaCl concentrations were studied: 0.87, 0.40 and 0.23 M.

## 3. Results and Discussion

The reflective substrate consisted of a 200 nm layer of Si_3_N_4_ over a silicon wafer. The surface was smooth and presented a strong reflectance at 400 nm, which was responsible for the violet color that the surface displayed; see Figure S1 (Supplementary Materials), so that any posterior deposit would increase the optical path of the whole set over the silicon substrate, shifting the surface color to longer wavelengths. Since this methodology requires a lower thickness to produce visual changes in the color, the control of thickness uniformity and, as a consequence, of color is easier to achieve. When DOPA was polymerized in dopamine-like conditions (pH 10 and absence of NaCl), there was no visual color change, which indicated the absence of poly-L-DOPA deposition.

### 3.1. Effect of NaCl Concentration on Film Growth and Color

Poly-L-DOPA coating over polyethylene, polyvinylidene difluoride and polytetrafluoroethylene substrates by the polymerization of DOPA in an aqueous buffer solution of low ionic strength has previously been reported [18]. However, when polymerization was carried out in a similar low ionic strength medium ([NaCl] = 0.1 M), only a subtle blue color was observed. Nevertheless, when polymerization was carried out at higher ionic strength ([NaCl] = 0.87 M), the blue color could easily be identified after two hours of polymerization, which is in agreement with the authors who claimed the importance of high ionic strength in the polymerization aqueous medium to achieve a successfully thick poly-L-DOPA coating [16].

**Figure 1:**
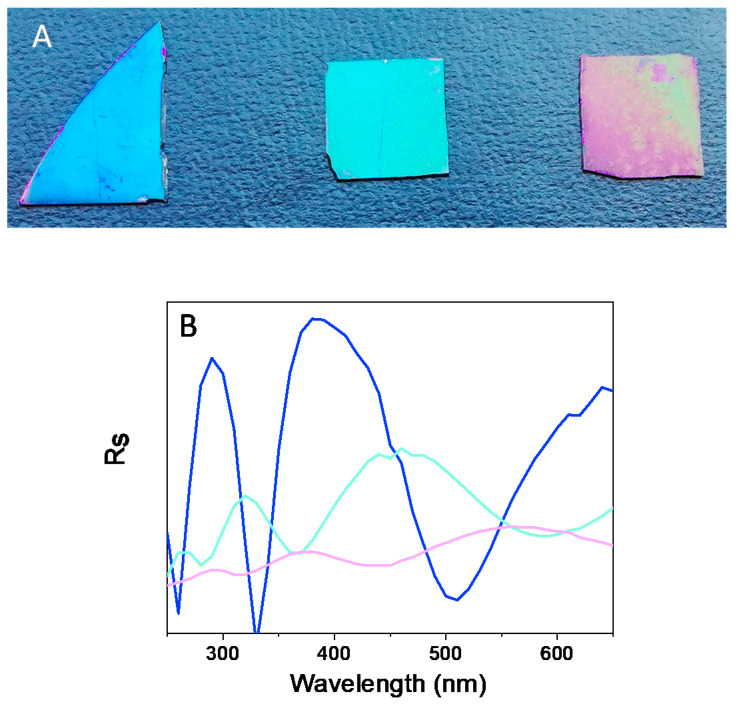
(**A**) Image of silicon nitride wafers after exposing the silicon nitride wafers for 9 h to the polymerization medium in the presence of different NaCl concentrations: 0.23, 0.40 and 0.87 M, from left to right. (**B**) Perpendicular reflectance (Rs) spectra at 70° of surfaces obtained with [NaCl] = 0.23 (blue), 0.40 (cyan) and 0.87 M (magenta).

Therefore, ionic strength seemed to be a determinant factor to obtain poly-L-DOPA coatings. First, the effect of NaCl concentration (in the polymerization medium) on both the morphology and color of poly-L-DOPA surfaces was evaluated. For this purpose, different NaCl concentrations in the polymerization medium were studied: 0.23, 0.40 and 0.87 M, while the polymerization time was chosen as 9 h in order to obtain a significant amount of deposit on the Si_3_N_4_ surface. The polymerization of L-DOPA (2 mg/mL) was carried out in an aqueous buffer solution (trizma buffer, pH = 8.5) that contained NaCl to increase the ionic strength. The deposition of poly(L-DOPA) on the silicon nitride wafer was carried out by submerging the silicon nitride wafer in an opened air-flask where the polymerization was taking place. When the concentration of NaCl was 0.23 M, the color of the surface changed to blue, and it progressively red shifted when the salt concentration was increased up to 0.87 M; see Figure 1A. The color uniformity was better for the two lower salt concentrations. However, for the highest concentration, areas with red and green coexisted, which visually indicated a different poly-L-DOPA thickness along the surface.

The experimental perpendicular component of the reflectance at 50° is shown in Figure 1B. The band responsible for the color was located around 390, 466 and 560 nm for the [NaCl] = 0.23, 0.40 and 0.87 M, respectively. It red shifted with the increase in salt concentration, which is in agreement with the visual perception of color.

The presence of a poly-L-DOPA deposit was confirmed by Raman spectroscopy. The Raman spectrum shows peaks at 1386 and 1530 cm^−1^, which have previously been reported in natural melanin samples [19]; Figure S2 (Supplementary Materials). The intensity of these peaks increased with the salt concentration, which indicated the formation of a thicker poly-L-DOPA film.

**Figure 2:**
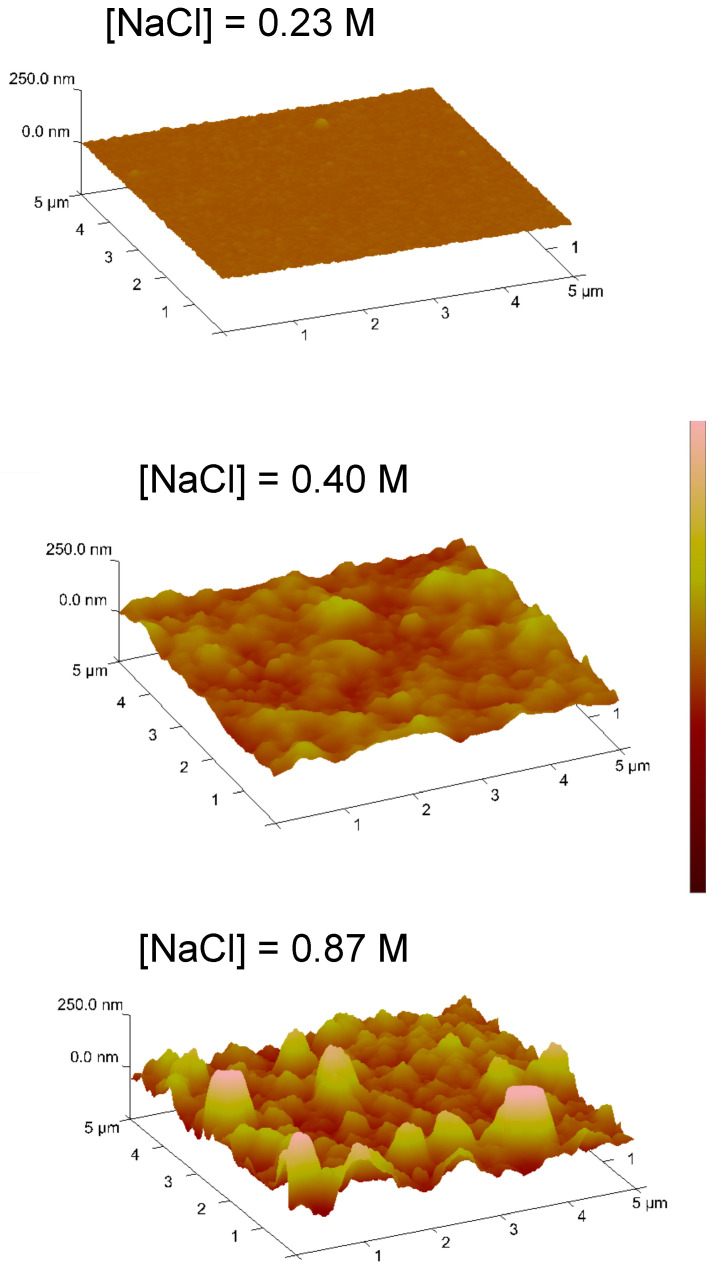
AFM images of surfaces after 9 h of polymerization and different NaCl concentrations in the polymerization medium.

A more in-depth analysis of the salt effect on the poly-L-DOPA coating characterization of surfaces was conducted by AFM. The poly-L-DOPA film thickness was calculated by making a needle-scratch on the surface and determining the depth of the scratch (thickness of film). Figure 2 shows the AFM images of surfaces obtained for all studied NaCl concentrations. When the NaCl concentration was 0.23 M, the surface presented a remarkably small roughness, the root mean square average roughness (*R_q_*) value was 3.4 nm and the thickness of the poly-L-DOPA coating was determined to be 8 ± 2 nm. Increasing the NaCl concentration to 0.57 M led to a rougher surface with a *Rq* of 27 nm and a thickness of 51 ± 27 nm. The surface obtained with the highest NaCl concentration presented a greater roughness value of 63 nm. It is important to highlight that the roughness value was always lower than the film thickness.

The roughness had a strong effect on the reflectance of the surfaces. As can be seen in Figure 1A, the increase in roughness decreased the intensity of the perpendicular component of the reflectance (*R_s_*), which agreed with the characterization of the roughness effect in polydopamine films [12].

### 3.2. Effect of Polymerization Time on Film Growth and Color

**Figure 3:**
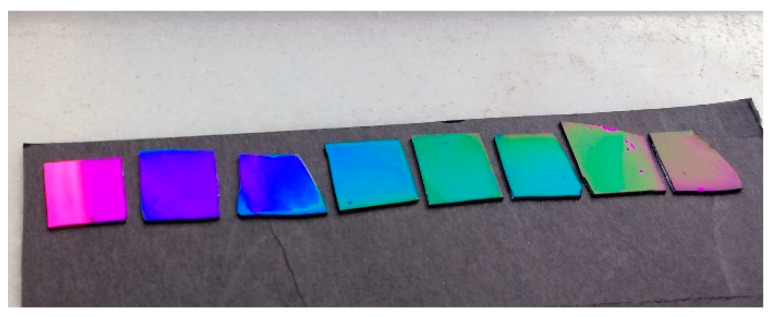
Image of silicon nitride surfaces after immersing the silicon nitride wafers in the polymerization medium for 2 to 8 h. The left surface corresponds to the bare silicon nitride surface.

Although it has been stated that an increase in the NaCl concentration decreases the quality of the coating surface, the extremely low growth rate forced us to choose a compromise between quality and simplicity of production. In this regard, we fixed the NaCl concentration at 0.87 M, and the polymerization time was varied from 2 to 8 h. The image of silicon nitride wafers upon modification with poly-L-DOPA at different times is shown in Figure 3. Thus, it could be seen that the color was notably modified from the initial violet color towards the red when the polymerization time was increased up to 8 h.

SEM and AFM were used to characterize the morphology of these surfaces. SEM images of the surface as a function of polymerization time are shown in Figure S3 (Supplementary Materials). It could be observed that the increase in the reaction time led to surfaces with a larger number and greater size of poly-L-DOPA aggregates. The presence of aggregates along the surface was more evident when the polymerization time exceeded four hours.

**Figure 4:**
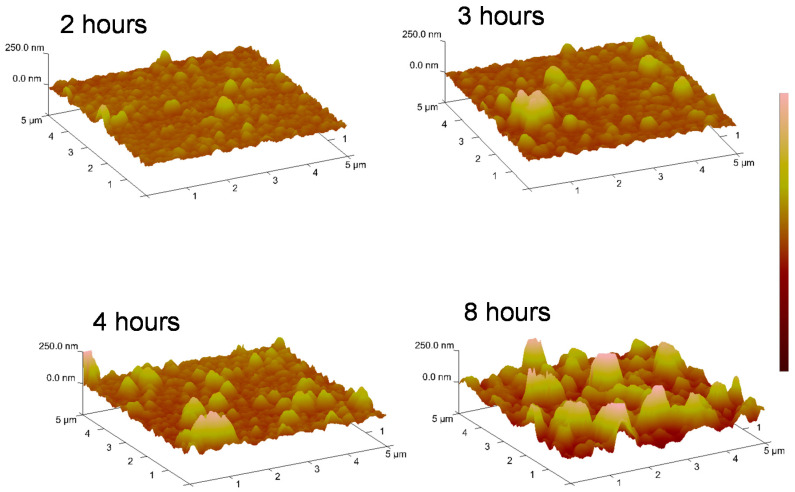
AFM images of surface topography after different polymerization times.

The effect of polymerization time on surface morphology could be observed more clearly in the AFM characterization. Figure 4 shows the AFM images of surfaces obtained at different polymerization times: 2, 3, 4 and 8 h. After 2 h, the surface seemed to be coated by a uniform granular cement. With the increase in polymerization time, a larger number of greater granules could be seen along the surface.

**Figure 5:**
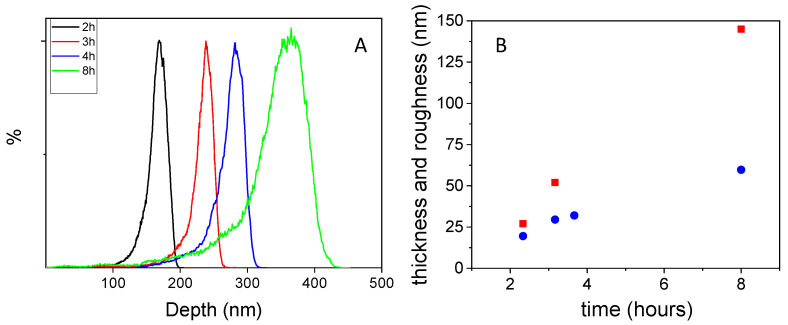
AFM characterization: (**A**) size of poly-L-DOPA aggregates as a function of time of polymerization; (**B**) roughness (■) and thickness (●) of poly-L-DOPA coating as a function of polymerization time.

The depth histograms are shown in Figure 5A to characterize the size distribution of the aggregates. The maximum of the depth histogram was 176 nm when the time of polymerization was 2 h. When the polymerization time was 4 h, the maximum of the depth histogram shifted to 282 nm. At 8 h, these aggregates had, notably, grown up to 365 nm. The *R_q_* values are shown in Figure 5B. The *R_q_* value increased with the polymerization time, from 20 nm for 2 h, up to 60 nm for 8 h of polymerization. Additionally, surface thicknesses of 28 and 52 nm were obtained after 2 and 3 h of polymerization, respectively. After 8 h of polymerization, the coating reached 145 nm thickness. Nevertheless, the roughness values were lower than the thicknesses in all polymerization times, unlike polydopamine coatings. This might indicate that, in the case of polydopamine film, growth is caused by the deposition of nanoparticles, while in the case of poly-L-DOPA, the deposition of oligomers might be the predominant mechanism.

To summarize, the poly-L-DOPA film deposited on silicon nitride surfaces by the oxidative polymerization of DOPA in the presence of NaCl 0.87 M led to colored surfaces from blue to magenta when the film thickness was in the range 28–145 nm and *R_q_* values in the 20–60 nm range. However, to achieve similarly colored surfaces by polydopamine coating, the thickness was reported to be in the 30–170 nm range, with the *R_q_* values nearly identical to the thickness values [12].

### 3.3. Optical Characterization: Refractive Index Determination

The determination of the refractive index of melanins has been a matter of study in the last two decades [20]. It has been reported that the refractive index of natural melanin from feathers of the birds-of-paradise (without isolating the melanin) in the visible wavelength range is in the 1.8–1.7 range (400–600 nm) [5]. On the other hand, the characterization of the refractive index of the synthetic eumelanin (from oxidative polymerization of DOPA) is barely reported [21]. To the best of our knowledge, only in [21] has the refractive index been determined by ellipsometry in silicon surfaces coated by a thick film of a mixture of synthetic melanin (from co-oxidation of dopamine and L-DOPA), and the reported values were 1.8–1.85 in the 400–600 nm range.

**Figure 6:**
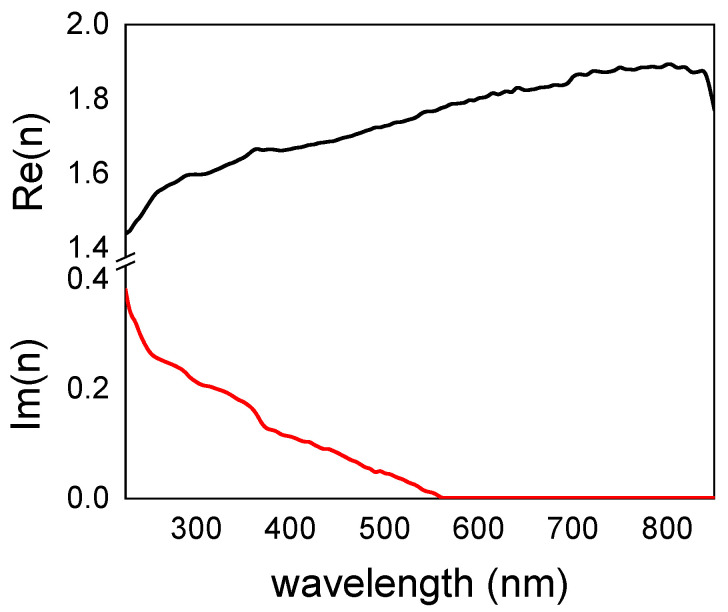
The real (**―**) and imaginary part (**―**) of the refractive index of the poly-L-DOPA as a function of the wavelength.

We used colored surfaces to determine the reliability of the characterized refractive index of poly-L-DOPA over a silicon substrate, since all optical properties, including color, are determined by the refractive index of the deposit. When the substance appears as a homogeneous thin layer, spectroscopic ellipsometry is the best tool. In order to achieve a greater accuracy, a thin and homogenous layer of poly-L-DOPA was grown on a silicon wafer, and eight spectra were taken at angles from 45 to 80° each 5°, so that we had data below and above the Brewster angle of silicon. The eight complex spectra of *ρ* = *R_p_*/*R_s_* were fitted simultaneously in a spectral range from 250 to 850 nm; see Figure 6. The spectra obtained from the fitting revealed that poly-L-DOPA presented a very high and nearly constant refractive index at Infrared (IR) wavelengths (*n* > 1.8 for *λ* > 650 nm). Right at the red spectral region (*λ* < 640 nm), the imaginary part of the refractive index became relevant, increasing its value for blue spectral regions. The refractive index decreased in the visible wavelength range (400–600 nm) gradually from 1.8 to 1.7. These values were slightly lower than the reported ones [21].

**Figure 7:**
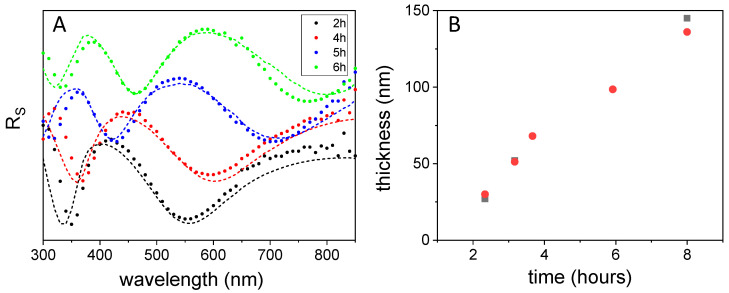
(**A**) Experimental (straight line) and fitted values (dotted) of perpendicular reflectance, R_s_, for surfaces obtained with different growth time at the incidence angle of 50°. (**B**) Optical (●) vs. AFM (■) thickness.

Knowledge of the refractive index allowed us to predict the optical response in different shapes as aqueous suspensions or thin film deposits. More specifically, we could reproduce the optical reflectance spectra by using the fitted data in a transfer matrix calculation [22] (Supplementary Materials). This procedure is able to determine both transmittance and reflectance both for parallel and perpendicular polarization and for any incidence angle. In Figure 7A, we plotted the experimental perpendicular component of the reflectance and its fitting for poly-L-DOPA coatings obtained at 2, 4, 5 and 6 h of polymerization. For the smallest thicknesses, agreement was remarkable, while for the thicker coating, optical data seemed to indicate that the real thickness is smaller than that estimated by AFM. If we compare the thicknesses of the different samples obtained by optical spectroscopy (Figure 7B) with those determined by AFM, we can conclude that there is a good agreement between them.

**Figure 8:**
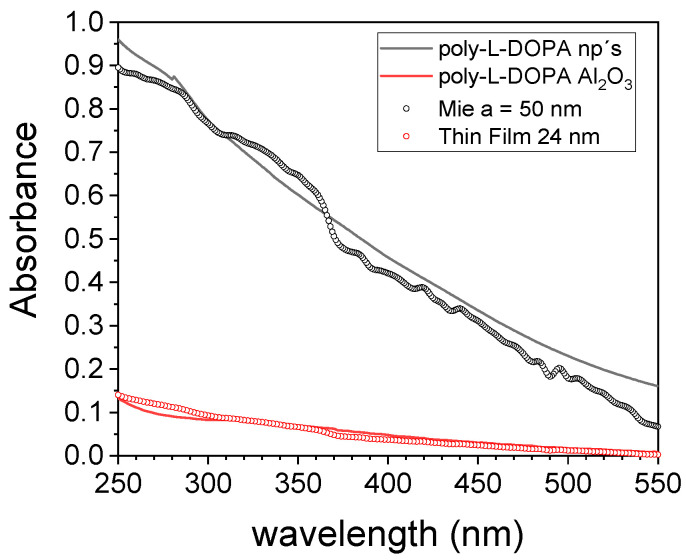
Absorbance spectra (line) and simulated spectra (empty circles) of the poly-L-DOPA film (red) and the aggregates of poly-L-DOPA aggregates in water (black).

The chemical composition and structure of both naturally occurring and synthetic melanins remains elusive [23,24]. This makes the rationalization of the UV-vis spectrum very complicated. However, the spectra for a dispersion of poly-L-DOPA aggregates in water as well as for a thin film were studied. For the UV-vis characterization of a pol-L-DOPA film, DOPA was polymerized on an alumina substrate, which was transparent in the UV-vis range. The UV-vis spectrum for the poly-L-DOPA film as well for the aggregates in water are presented in Figure 8.

The aggregates of poly-L-DOPA in water presented a broad declining absorbance with the wavelength in the 200–800 nm range without any spectral characteristic in agreement with prior works dealing with synthetic melanins [25], but a broad and weak absorption at 280 nm, which could be attributed to the initial precursor (DOPA) of the polymerization, was observed. The spectrum profile resembled the scattering profile, which presented an intensity dependence of λ^−4^. The experimental spectrum was simulated taking into account the Mie theory [26,27] by considering a diameter of 50 nm for the aggregates; see Figure 8. On the other hand, in the spectrum of the poly-L-DOPA film, two broad bands could be detected; see Figure S4 (Supplementary Materials): the first, from 290 to 425 nm and centered around 350 nm, and a second broad band above 500 nm. The band between 298 and 425 nm could be attributed to the absorption of 5,6-dihydroxyindole-2-carboxylic acid (DHICA) and its π-stacked forms [28,29]; DHICA had been identified as an intermediate in the synthetic pathways for eumelanin [30]. The less energetic band could be due to different redox products of DHICA and their π-stacked forms. The experimental spectrum was fitted according to the theoretical equation for the absorption coefficient, *α = 4πk/λ* [2]; the fittings are shown in Figure 8.

## 4. Conclusions

New colored poly-L DOPA surfaces were prepared. Poly-L-DOPA was deposited on a reflective silicon nitride substrate by immersing the wafer in the DOPA polymerization medium (pH = 8.5) that had a high ionic strength. The use of a reflective substrate permitted us to obtain visual information about the coating process and thickness uniformity of the deposited poly-L-DOPA film. An ionic strength was necessary to achieve the substrate coating. When the concentration of NaCl was 0.23 M, a high-quality surface was obtained with a roughness of 6 nm. An increase in ionic strength led to an increase in film thickness and surface roughness.

For a NaCl concentration of 0.87 M, the control of the polymerization time permitted us to control the film growth; colors changed from blue to magenta when the film thickness was increased from 28 to 104 nm. The *R_q_* values were in the 20–60 nm range. The roughness values were lower than the thicknesses at all polymerization times, which may indicate that during film growth, the poly-L-DOPA deposition of oligomers may be the predominant mechanism.

These surfaces, which were uniform in color and concomitant in thickness, were used to determine the refractive index of poly-L-DOPA by ellipsometry spectroscopy. Poly-L-DOPA presented a very high and nearly constant refractive index at IR wavelengths (*n* > 1.8 for *λ* > 650 nm). The imaginary part of the refractive index become relevant at the red spectral region (*λ* < 640 nm). The refractive index in the visible wavelength range (400–600 nm) gradually ranged from 1.7 to 1.8.

The refractive index obtained in this work permitted the reproduction of the reflectance spectra of surfaces, the absorbance spectra for poly-L-DOPA film, and poly-L-aggregates dispersed in solution.

The newly colored poly-L DOPA surfaces have great potential for the development of future photonic materials. Poly-L-DOPA presents a higher chemical versatility than polydopamine. In addition to the polydopamine chemistry, a poly-L-DOPA coating is rich in carboxylic acids, and new materials may be obtained from these new surfaces.

## Data Availability

The data that support the findings of this study are available from the corresponding authors upon reasonable request.

## Supplementary Materials

The following are available online at https://www.mdpi.com/article/10.3390/nano11092320/s1, Figure S1: Left) SEM image of the substrate: 200 nm of a silicon nitride layer deposited over silicon. Right) Image of the surface color, Figure S2: Raman spectra of silicon nitride substrate (violet), silicon nitride coated with poly-L-dopa, Figure S3: SEM images of surface after being coated with poly(L-dopa) at different polymerization times, Figure S4: Absorbance spectra of the poly-L-dopa film and the aggregates of poly-L-dopa aggregates in water, Transfer matrix calculation.
