# Supplementary material for: Colored Surfaces Made of Synthetic Eumelanin
Source: Nanomaterials (Basel). 2021 Sep 7;11(9):2320. doi: 10.3390/nano11092320 (PMC8468714; doi:10.3390/nano11092320)
Supplement: Supplementary file 1 [file nanomaterials-11-02320-s001.zip › nanomaterials-1342470-supplementary.pdf]

nanomaterials

Supplementary Materials

Colored surfaces made of synthetic eumelanin

Gema Marcelo [1,2,*], María del Mar López-González [3], Milena Vega [4] and Carlos Pecharromán [5,*]

1 Departamento de Química Analítica, Química Física e Ingeniería Química, Universidad de Alcalá, 28805 Alcalá de Henares, Madrid, Spain
2 Instituto de Investigación Química "Andrés M. Del Rio" (IQAR), Universidad de Alcalá, 28805 Alcalá de Henares, Madrid, Spain
3 Instituto de Ciencia y Tecnología de Polímeros (CTP, CSIC), C/Juan de la Cierva 3, 28006 Madrid, Spain; mar@ictp.csic.es (M.L.G.)
4 Departamento de Ingeniería Química y Textil, Pl/La Merced s/n 37008 Universidad de Salamanca, Salamanca, Spain; mvega@usal.es (M.V.)
5 Instituto de Ciencia de los Materiales de Madrid (ICMM, CSIC), C/Sor Juana Inés de la Cruz 3, 28049 Madrid, Spain
* Correspondence: gema.marcelo@uah.es (G.M.); cpg@icmm.csic.es (C.P.)

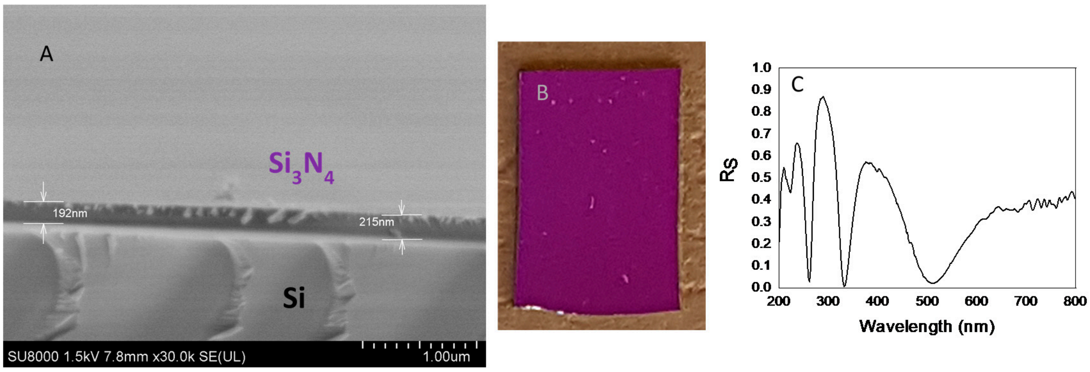

Figure S1. (**A**) SEM image of the substrate: 200 nm of a silicon nitride layer deposited over silicon. (**B**) Image of the surface color. (**C**) perpendicular reflectance, R_s, of the silicon nitride surface at the incidence angle of 50°.

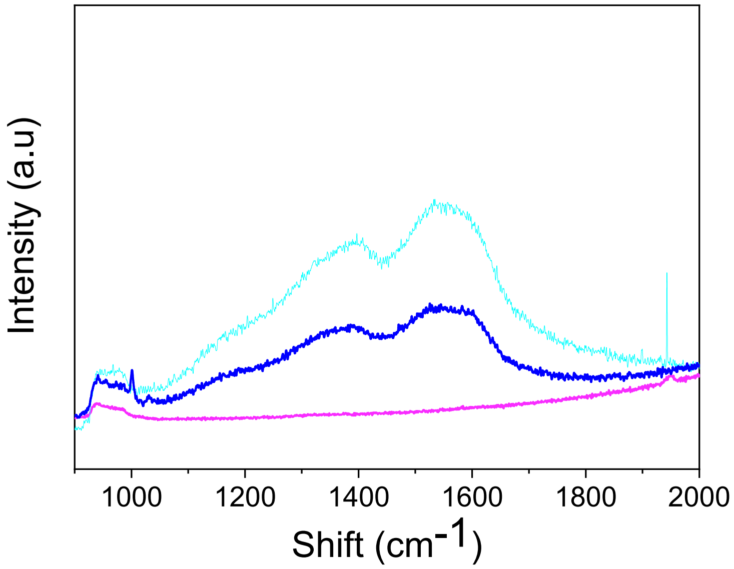

Figure S2. Raman spectra of silicon nitride substrate (violet), silicon nitride coated with poly-L-dopa: NaCl= 0.23 M (blue) and 0.40 M (cyan).

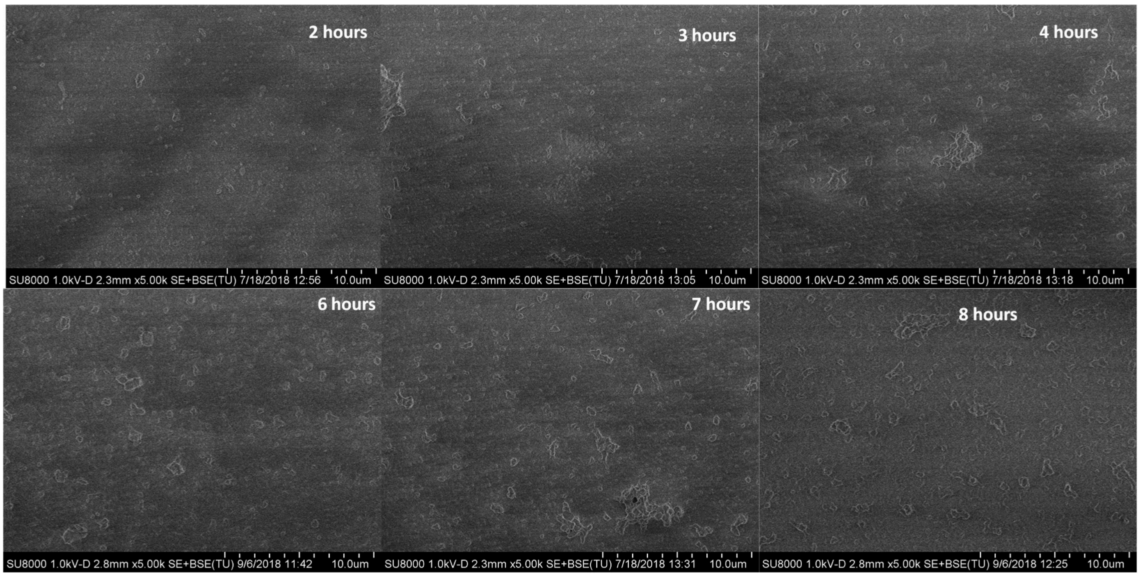

Figure S3. SEM images of surface after being coated with poly(L-dopa) at different polymerization times.

Optical Characterization

Interferential phenomenon is being used to determine the thickness and refractive of thin layers by fitting ellipsometry measurements to theoretical calculations. The equations that rule this phenomena are known as Fresnel coefficients applied to multilayers. The general theory is a variation of the "Transfer Matrix Procedure" and was developed by Abèles M. Born and Wolf [1]. Basically, the method states that each layer is represented by a 2 × 2 matrix whose elements depend on the thickness, refractive indices of substrate and layer as well as the incidence angle and polarization state, so that the reflectance of the whole structure, for any incidence or polarization angle, can be deduced from the matrix product of all the layers.

The reflectance of a simple structure with a thin layer on a substrate can be visualized by only considering a simple interferential phenomenon. In this sense, when the summation of the optical path of the layers (defined as the product of the thickness times the refractive index) is a semi-integer of the incident wavevector, there is a maximum on the R_s component of the reflectance, according to Equation (1).

$$\begin{cases} l\dfrac{\pi}{2} = \dfrac{2\pi}{\lambda_0}\sum_j n_j d_j \cos(\theta_j) & \text{minimum condition} \\ m\pi = \dfrac{2\pi}{\lambda_0}\sum_j n_j d_j \cos(\theta_j) & \text{maximum condition} \end{cases}, \quad (1)$$

Where n_j, d_j and q_j are respectively the refractive index, thickness and propagation angle corresponding to the *j* layer and *l* and *m* integers. Although criteria of Equation (1) can be fulfilled at different incident angles for a given wavelength, we are only interested into the maximum condition for reflectance according to the human visual perception. Visual reflection is seldom verified at normal incidence so that, we need to consider better grazing angles. Moreover, the intensity of the perpendicular component (R_s) increases for larger incident angles, while the parallel component (R_p) tends to cancel around the Brewster condition, which for a Si_3N_4 layer over a Si substrate is satisfied at incidence angles from 60° to 70°. Therefore, it can be concluded that, the color perception can be modeled approximately by the perpendicular polarization component of the reflection at a large incidence angle (q_i > 50°). In this sense, the dominant color wavelength is given by the following maximum condition (Equation 2):

$$\lambda = \frac{m}{2}\left[n_{melanin}d_{melanin}\cos\left(\arcsin\left(\frac{\sin\theta_i}{n_{melanin}}\right)\right) + n_{\mathrm{Si3N4}}d_{\mathrm{Si3N4}}\cos\left(\arcsin\left(\frac{\sin\theta_i}{n_{\mathrm{Si3N4}}}\right)\right)\right] \quad (2)$$

This expression, which is an expansion of Equation 1 (maximum condition), basically states that the sum of the optical path along both layer (Si_3N_4 substrate and melanin layer) Where m is the reflection order (m = 2 for our samples) and q_i the incidence angle. In our case, we have chosen a large incidence angle (q_i = 50°) for experimental considerations.

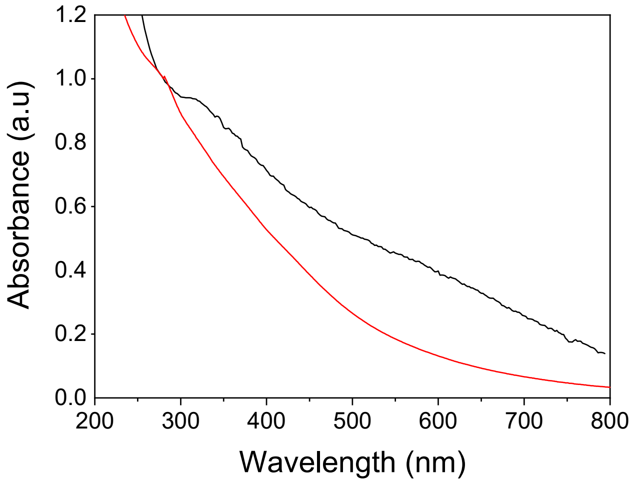

Figure S4. Absorbance spectra of the poly-L-dopa film (—) and the aggregates of poly-L-dopa aggregates in water (—). Absorbance was normalized at 280 nm.

Reference

1. Born, M., and Emil Wolf. Principles of Optics; Electromagnetic Theory of Propagation, Interference, and Diffraction of Light. Pergamon Press: Oxford, 1964.
